# Unusual Presentation of Extraskeletal Mesenchymal Chondrosarcoma: A Case Report

**DOI:** 10.7759/cureus.45974

**Published:** 2023-09-26

**Authors:** Mathilde Bernard, Ramy Samargandi

**Affiliations:** 1 Orthopedic Surgery Department, Centre Hospitalier Régional Universitaire (CHRU) de Tours, Tours, FRA; 2 Orthopedic Surgery Department, Faculty of Medicine, University of Jeddah, Jeddah, SAU

**Keywords:** mass resection, sarcoma soft tissue, soft-tissue tumor, chondrosarcoma, extraskeletal mesenchymal chondrosarcoma, mesenchymal chondrosarcomas

## Abstract

Mesenchymal chondrosarcomas are extremely rare and aggressive tumors that primarily affect patients between the ages of 20 and 30. These neoplasms are typically found in the lower limbs and cranial region. Their occurrence within soft tissues is exceedingly rare, and the initial presentation often includes immediate metastatic dissemination. Given the extraordinarily low prevalence of extraskeletal mesenchymal chondrosarcoma, treatment approaches remain non-standardized. Surgical resection combined with neoadjuvant chemotherapy or radiotherapy is the most commonly favored strategy by medical teams. In this case report, we present the case of a 72-year-old patient with no specific medical history, who presented with a non-metastatic extraskeletal mesenchymal chondrosarcoma located in the popliteal fossa. The therapeutic intervention encompassed surgical resection followed by adjuvant radiotherapy. After 18 months of follow-up period, there was no evidence of local recurrence or distant metastases. The disparity between the patient's clinical characteristics and the existing medical literature may provide new insights into understanding this neoplastic entity.

## Introduction

Mesenchymal chondrosarcoma is an extremely rare, highly malignant tumor, accounting for less than 10% of all chondrosarcomas. It usually involves bone (particularly axial skeleton and femur) [1] but can approximately occur in extra-skeletal localization in 30% [2-4]. The most common sites of extraskeletal mesenchymal chondrosarcoma (EMC) are the cephalic extremities (including the nervous central system and orbit) and lower limb [3,5]. Few cases are described in other locations like the kidney, pancreas, uterus, and heart [2,5-8]. Sex ratio appears to vary, with some studies showing a slight predominance of women [3,4], while others indicate a higher proportion of men [1]. These are aggressive tumors with a high metastatic potential and a poor prognosis in the presence of secondary localization [1]. Patients affected by this condition are typically young (20-30 years old). The diagnosis is established through imaging modalities, such as computerized tomography (CT) scans and magnetic resonance imaging (MRI), as well as histopathological assessments, which may involve immunohistochemistry. Treatment approaches are not yet standardized but typically include wide surgical resection, chemotherapy, and radiation therapy tailored to individual cases. There are a few studies reporting extraskeletal location in the lower extremities [9,10]. In this article, we present the case of an elderly patient with an extraskeletal, non-metastatic localization. A brief literature review will be provided in the second part of the article.

## Case presentation

A 72-year-old female patient, with an unremarkable medical history or prior treatments, presented with a painless soft tissue mass of the posterior region of the distal thigh, which increased progressively in size. She was referred by the radiologist who discovered the lesion to the multidisciplinary tumor board meeting. Physical examination revealed a palpable, mildly tender, firm, and immobile mass in the posterior portion of the distal thigh region, with normal overlying skin. No other palpable masses were found in other sites, and there were no abnormalities in the neurological and vascular examinations. Other general and systemic examinations were normal, and the results of the blood workup were unremarkable.

A CT scan and MRI were performed in December 2021, revealing a deep soft tissue mass measuring 7 cm in its longest dimension in the posterior region of the distal thigh.

**Figure 1:**
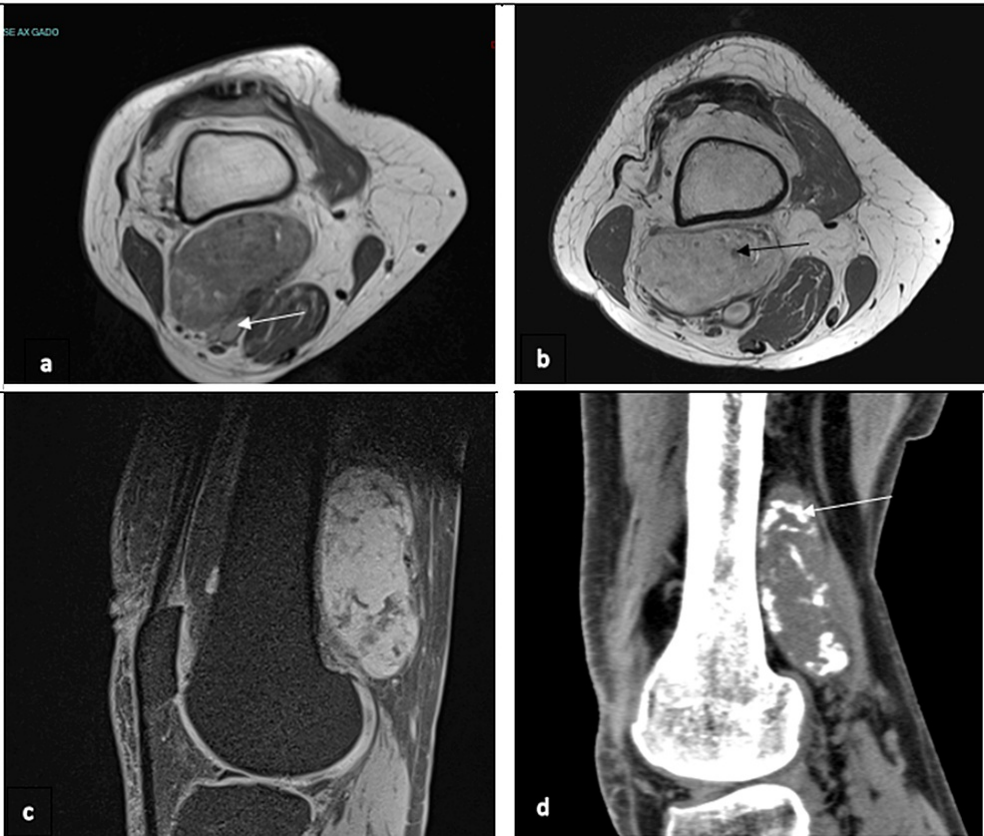
Imaging of the lesion at diagnosis a. Preoperative MRI  with axial T1-weighted image with the lesion adjacent to the popliteal pedicle (white arrow); b. Axial T1-weighted gadolinium-enhanced image, showing calcifications (black arrow); c. Sagittal T1-weighted fat-saturated image after gadolinium injection shows heterogeneous uptake, with some areas of low signal intensity indicating calcification; d. Sagittal CT scan demonstrating calcifications of the lesion (white arrow)

Regarding the MRI findings (Figures 1A-1C), they revealed a heterogeneous mass with low signal intensity on the T1-weighted image and high signal intensity on the T2-weighted image, along with significant contrast enhancement following gadolinium injection. On the CT scan, the lesion exhibited calcification (Figure 1D).The

The differential diagnosis included calcified soft tissue lesions such as extraskeletal osteosarcoma, extraskeletal myxoid chondrosarcoma, synovial sarcoma, myositis ossificans, hematoma, hemangioma, and calcific myonecrosis [11]. Because of this potential differential diagnosis, a biopsy was recommended.

**Figure 2:**
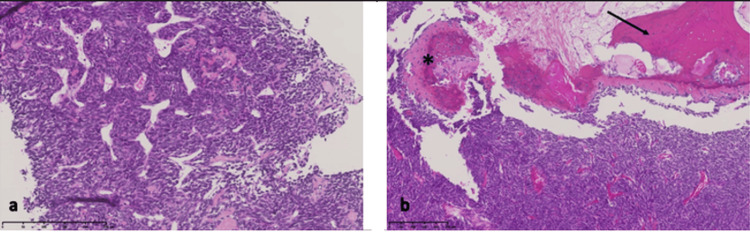
Histological features of the tumor a. On a microbiopsy, the tumor was composed of monotonous spindle cells with a staghorn vascular pattern; b. The resection specimen of the tumor showed an admixture of monotonous spindle or round cells, cartilage islands (asterisk), and bone (black arrow)

In January 2022, an ultrasound-guided biopsy was performed, revealing a tumor characterized by monotonous spindle cells with a high N: C ratio associated with a staghorn vascular pattern (Figure 2A). Immunohistochemistry did not reveal any expression of S100, STAT6, SS18, CD34, epithelial markers (cytokeratins, epithelial membrane antigen), or myogenic markers (actin, desmin, h-Caldesmon). An RNAseq analysis identified a HEY1::NCOA2 fusion transcript, allowing pathologists to diagnose mesenchymal chondrosarcoma.

The patient's case was discussed in the multidisciplinary tumor board meeting, and it was decided to conduct an extension assessment for staging with cerebral MRI and thoracoabdominal-pelvic computed tomography (TAP-CT), followed by performing a wide surgical resection with short margins around the popliteal vessels and then followed by adjuvant radiotherapy.

The staging assessment did not reveal any metastases. The surgical procedure took place in March 2022. Macroscopy examination revealed a firm tumor measuring 6.5 cm and containing calcified areas. Pathological examination confirmed the presence of a tumor composed of round or spindle cells with a hemangiopericytomatous vascular pattern admixed with bone tissue areas and only focal islands of cartilage (Figure 2B). The resection was complete with negative margins.

The patient received adjuvant radiotherapy that covered the surgical field using the external beam radiation therapy (EBRT) technique (60 Gy in 30 fractions), which was administered five weeks postoperatively

A surveillance plan was established, consisting of contrast-enhanced MRI combined with thoracic CT scans, alternated with TAP (thorax, abdomen, and pelvis) CT scans every four months for a period of three years, then biannually until five years postoperatively.

In 18 months of postoperative follow-up, there have been no signs of local recurrence or distant metastasis.

## Discussion

EMC is an extremely rare malignant tumor and represents less than 1% of all chondrosarcomas [5]. The lesions primarily affect the lower limbs and cephalic region [3]. Typical age is between 20 and 30 years [1,3,12]. A case of a nine-year-old child with a spinal location and no recurrence after a nine-year follow-up has also been described in the literature [13]. Our case involves a 70-year-old lady, which is not academic. Only a few cases of individuals over 70 years old have been described in some series [3].

Due to the high degree of malignancy, it is not uncommon for the clinical presentation to be aggressive, with metastasis evident from the outset. The lesions are primarily found in the lungs [1,3,5]. The origin and localization of the primary lesion do not appear to have an impact on survival. In the absence of metastasis, the average survival after diagnosis is approximately 20 years [1]. However, the presence of metastasis remains the primary factor for poor prognosis, leading to impaired survival [3]. Seventeen months after resection and 20 months after the first presentation, the patient showed no signs of recurrence or secondary lesions.

Several atypical locations have been described in the literature, including the uterus, orbit, kidney, or pancreas [2,5-7]. Localization in the lower limbs is one of the more common sites for this type of tumor. However, its proximity to the popliteal pedicle made excision challenging, necessitating resection with narrow margins and subsequent adjuvant therapy.

Characteristic imaging reveals a tissue mass with calcifications [3,4]. At this stage, the main differential diagnoses include calcified tumors such as synovial sarcoma, extraskeletal osteosarcoma, extraskeletal myxoid chondrosarcoma, myositis ossificans, and vascular tumors. Synovial sarcoma is mostly found in the popliteal fossa, just like our case, but in young adults. It’s a multilobulated lesion with eccentric calcification. The lesion had marked heterogenous enhancement after gadolinium injection in MRI [14,15]. Myositis ossificans is a solitary calcified lesion that is found in skeletal muscle. Traumatic history is found most of the time. These lesions can also be confounded with soft tissue osteosarcoma [14]. Soft tissue osteosarcoma is a well-circumscribed heterogeneous mass with dense mineralization. The lesion appears heterogeneous after a gadolinium injection [16].

Histologically, the tumor is usually characterized by the presence of small round cells with a staghorn vasculature and a variable proportion of islands of well-differentiated cartilage [3,5,6,17,18]. Immunohistochemistry may show positivity of S100, SOX9, or CD99 by the tumor cells [3,4]. Most mesenchymal chondrosarcomas harbor a HEY1::NCOA2 rearrangement, and an IRF2BP2::CDX1 rearrangement has also been reported [1,19]. In cases of central nervous system localization, it may be useful to search for INI-1, which will be negative in mesenchymal chondrosarcoma but positive in teratogenic rhabdoid tumors, myoepithelial carcinoma, or extraskeletal myxoid chondrosarcoma [3]. The main histological differential diagnoses include Ewing sarcoma, due to the presence of small round mesenchymal cells [17], synovial sarcomas, and malignant solitary fibrous tumors.

Regarding treatment, the gold standard remains wide surgical resection. Adjuvant chemotherapy, such as doxorubicin is recommended by most teams for mesenchymal chondrosarcoma involving bone. However, there is no standardized protocol, specifically when presenting in soft tissue [1,3,4,18]. Some teams may recommend radiotherapy or chemotherapy alone if the mass is not resectable [17]. In cases of positive margins, radiotherapy can be used locally, although chemotherapy is generally more effective [12]. Radiotherapy may increase recurrence-free survival, according to the team led by Kawaguchi [20]. Due to the patient's age of 72 years and the risk of side effects and intolerance, chemotherapy was not offered. Since the resection was in close proximity to the popliteal vessels with narrow margins, radiotherapy was recommended to improve local control, resulting in no recurrence since the last follow-up.

## Conclusions

A mesenchymal extra-articular chondrosarcoma is an extremely rare tumor, typically found in young individuals. This case, presenting with a tumor localized in the popliteal fossa, non-metastatic in a 72-year-old patient, and showing no recurrence after treatment, is highly unusual. The treatment approach, which lacks consensus, often involves resection with adjuvant chemotherapy. In our case, given the advanced age of the patient, resection with adjuvant radiotherapy was chosen to optimize treatment tolerance. The most recent follow-up, 18 months after treatment, demonstrates an absence of recurrence. These atypical findings, in light of the existing literature, could contribute to a better understanding of the characteristics of this rare tumor.
